# Prevalence of congenital malformations at the “les Orangers” maternity and reproductive health Hospital of Rabat: descriptive study of 470 anomalies

**DOI:** 10.1186/s12887-020-02179-6

**Published:** 2020-06-03

**Authors:** K. Forci, M. H. Alami, E. Bouaiti, M. Slaoui, A. Mdaghri Alaoui, A. Thimou Izgua

**Affiliations:** 1 Congenital anomalies research team, Faculty of Medicine and Pharmacy of Rabat, University Mohamed V Rabat, B.P: 174 Downtown-Rabat, Rabat, Morocco; 2 “Les Orangers” Maternity and Reproductive Health Hospital of Rabat, CHU IBN SINA, Rabat, Morocco; 3 Laboratory of biostatistics, clinical & epidemiological research, Faculty of Medicine and Pharmacy of Rabat, University Mohamed V, Rabat, Morocco; 4 Faculty of Medicine and Pharmacy of Rabat, University Mohamed V, Rabat, Morocco; 5 Dysmorphology Unit and Congenital Anomalies, Pediatric Department 2, HER, CHU IBN SINA, Rabat, Morocco; 6 Center of consultations and external explorations, HER, CHU IBN SINA, Rabat, Morocco

**Keywords:** Congenital malformation, Prevalence, Association, Antenatal diagnosis, Morocco

## Abstract

**Background:**

Congenital malformations are described in about 3% of live births and 20% of stillbirths in the industrialized countries.

The prevalence of congenital anomalies in developing countries, including Morocco, is not well known at the national level.

The aim of our study is to conduct a descriptive exploratory analysis of congenital malformations cases diagnosed at the “Les Orangers” Maternity and Reproductive Health Hospital in Rabat.

**Methods:**

We collected all the cases of congenital malformations diagnosed at the “Les Orangers” Maternity and Reproductive Health Hospital in Rabat, from January 1st, 2011 to June 31st, 2016.

Data were reported on pre-established sheets and on a registry of malformations. Total and specific prevalences were calculated for each malformation. A principal component analysis (PCA) was then conducted followed by a Varimax rotation in order to identify the different associations of malformations in our series.

**Results:**

We registred 245 cases of congenital malformations out of a total of 43,923 recorded births; a prevalence of 5.58 per thousand births of which 19.2% were FDIU (fetal deaths in utero).

A polymalformative syndrome was found in 26.5% of cases which makes a total number of 470 anomalies. The musculoskeletal anomalies predominate with a rate of 33%, followed by neurological abnormalities 18%, of whom 31% were hydrocephalus, 26.2% anencephaly, and 20.24% spina bifida. Malformations of the eye, ear, face and neck were described in 12% of the cases, while genetic abnormalities were observed in 8,5% of which 87.5% represented Down syndrome.

The antenatal diagnosis of congenital malformations was performed in 28.6% of cases.

**Conclusions:**

Our study provides a general overview of the epidemiological situation related to different types of congenital anomalies for a specific area in Morocco.

It represents a database that should be complemented by other multicenter studies and the implementation of a national registry to determine the prevalence of congenital malformations at a national level.

## Background

Malformations are often described as congenital defects whether they are diagnosed at birth or later. Thus, the real defects or “primary defects” should be distinguished from distortions and disruptions that are secondary to an extrinsic factor and called “secondary defects”. This distinction is essential to establishing genetic counseling, as it allows evaluating the risk of malformation occurrence or recurrence and proposing appropriate prevention.

A congenital malformation (CM) is a morphological abnormality that results from an abnormal development process during the formation of the embryo or fetus. Depending on their types, locations and sizes, malformations can cause functional, psychological, or aesthetic disorders that presently affect more than 8 out of every 1000 children [1, 2].

Congenital malformations account for about 3% of live births and 20% of stillbirths. In industrialized countries they represent a frequent cause of infant mortality, morbidity and disability [3–6].

According to the WHO (World Health Organization), about half of congenital anomalies cannot be attributed to a specific cause. Some risk factors or causes are often associated and there are three etiological groups: [7].

- Genetic intrinsic causes (10–15%): chromosomal, genetic or epigenetic;
- Extrinsic environmental causes (10–15%): infectious agents (rubella, toxoplasmosis, cytomegalovirus), physical (ionizing radiation, hyperthermia), medicinal (isotretinoin, thalidomide, antiepileptics), metabolic (maternal diabetes, folic acid deficiency), toxic (tobacco, fetal alcohol syndrome), mechanical (oligoamnios);
- Multifactorial causes (20–25%)

Congenital malformations are one of the leading causes of morbidity and neonatal mortality in all developing countries in general and in Morocco in particular. According to the results of the last National Survey of Population and Family Health (ENPSF) 2017–2018, the neonatal mortality rate in Morocco is 13.56 per thousand live births with an infant mortality rate (less than 1 year) of 18% [8]. Unfortunately, the prevalence of congenital malformations and their causes are not yet established at the national level due to the lack of a nation-wide congenital malformations monitoring system.

Consequently, the aim of the present study is to conduct a descriptive exploratory analysis of CM cases diagnosed at “Les Orangers” Maternity and Reproductive Health Hospital in Rabat.

## Methods

### Study design

This is a descriptive epidemiological study of CM cases diagnosed prenatally by obstetrical ultrasound and / or at birth during a routine clinical examination of all newborn infants in the “Les Orangers” Maternity Hospital and Reproductive Health of Rabat, Morocco during the period from January 1st, 2011 to June 31st, 2016.

This hospital is a level 3-facility with an average annual delivery rate of 8000. It receives parturient/laboring women from all over Morocco. In addition, it ensures the monitoring of high risk or as well as normal pregnancies by carrying out antenatal checkups and obstetric ultrasounds.

All pregnant women whose fetuses or newborns had a malformation were included, regardless of the term or outcome of the pregnancy.

Cases where the malformation was suspected on obstetrical ultrasound but not confirmed at birth were excluded as were the cases of refusal to participate in the survey.

Parents were informed about the terms and objectives of the study and their consent was obtained. A significant proportion of women included in our study were illiterate and for cultural reasons they prefer to give an oral agreement without signing. At least 3 persons from the medical have witnessed each oral consent. Furthermore, oral explanations and a written document describing the information relevant to their consent and the contact details of the researcher were provided to each patient. The ethics committee accepted this procedure.

### Data collection

The collection of information was carried out by a doctor and reported on pre-established sheets and on “Les Orangers” Maternity’s Malformations Register.

To determine the prevalence, we opted for the international definitions used by EUROCAT and the ICBDSR:

- Total prevalence: total number of cases (live births, stillbirths and medical interruptions of pregnancy) divided by the total number of births (live births and stillbirths).
- Prevalence of live births: number of children born alive with the anomaly divided by the total number of live births [9, 10].

To classify the malformations we used the following definitions of malformation types:

- “Isolated” malformation: any malformation not associated with a chromosomal abnormality or other major abnormality of another system.
- Polymalformations: when there is an association of at least two malformations. They can correspond to three different situations [11]:·
  1. A sequence: is a set of anomalies resulting from a single anomaly or a mechanical factor: For example the oligoamnios sequence or Potter’s sequence, following a lack of amniotic fluid.
  2. A malformative syndrome: is a set of unrelated abnormalities, all derived from the same cause and not corresponding to a sequence, e.g., Down syndrome.
  3. An association: is the unplanned occurrence of at least two unrecognized malformations as a sequence or syndrome, e.g., VACTER syndrome, acronym for “vertebral, anal, cardiac, tracheal, esophageal, radial and / or renal malformations”.

### Statistical analysis

Data entry and statistical analysis was carried out using SPSS.18.0 software. Total and specific prevalences were calculated for each malformation. Principal component analysis (PCA) followed by Varimax rotation was performed. The selected factors had a true value greater than 1.25. The items retained within each factor had a value greater than or equal to 0.40 within the factor and a value below 0.40 within the other factors. A forced structure with three inter-correlated factors was selected for each malformation.

## Results

During the study period, 43,923 births were included, of which 245 cases had one or more congenital malformations, which represents a prevalence of 5.58 per thousand births (or 55.8 per 10,000 births). Of these cases, 96.7% had one or more clinically visible malformations, 51.9% were males and 43.5% were females with a sex ratio of 1.19.

A polymalformative syndrome was found in 26.5% of cases which explains why the total number of identified congenital malformations, with 470 anomalies, exceeds the number of patients.

Within these 470 reported CMs, musculoskeletal malformations accounted for 33% of cases followed by neurological malformations with a rate of 18%, and then abnormalities of the eye, ear, face and neck at 12%. Genetic abnormalities represented 8.5%. (The different types and subtypes of recorded malformations are summarized in Tables 1 and 2).

**Table 1:** The different types of congenital malformations according to the International Classification ICD-10 [12]

Types of congenital malformations according to the International Classification ICD-10	Prevalence compared to all malformations in percentage
**Q00-Q07** Congenital malformations of the nervous system	18
**Q10-Q18** Congenital malformations of eye, ear, face and neck	12
**Q20-Q28** Congenital malformations of the circulatory system	8
**Q30-Q34** Congenital malformations of the respiratory system	1
**Q35-Q37** Cleft lip and cleft palate	4,5
**Q38-Q45** Other congenital malformations of the digestive system	4
**Q50-Q64** Congenital malformations of genital organs	7,5
**Q65-Q79** Congenital malformations and deformations of the musculoskeletal system	33
**Q80-Q89** Other congenital malformations	4
**Q90-Q99** Chromosomal abnormalities, not elsewhere classified	8,5

International Statistical Classification of Diseases and Related Health Problems 10th Revision (ICD-10)-WHO Version for; 2017

**Table 2:** Prevalence of types and subtypes of congenital malformations identified at “Les Orangers” Maternity and Reproductive Health Hospital of Rabat, Morocco

Types and subtypes of congenital malformations according to classification (ICD-10)	No./470	%	Total prevalence per 10,000 live births
**Q00-Q07 Congenital malformations of the nervous system**			
Hydrocephalus (Q03.9)	26	31	5,9
Anencephaly (Q00)	22	26,2	5
Spina-bifida (Q05.9)	17	20,24	3,8
Encephalocele (Q01.9)	5	6	1,14
Microcephaly (Q02)	4	4,76	0,9
Holoprosencephaly (Q04.2)	4	4,76	0,9
Dandy Walker syndrome (Q03.1)	2	2,4	0,45
Arnold Chiari syndrome (Q07.0)	1	1,2	0,2
Facial paralysis (Q07.8)	1	1,2	0,2
Agenesis of corpus callosum (Q04.0)	1	1,2	0,2
Absence of Thalami^a^	1	1,2	0,2
Total	84	100.00	19
**Q10-Q18 Congenital malformations of the eye, ear, face & neck**			
Craniofacial dysmorphism^a^	29	51,78	6,6
Short neck^a^	6	10,7	1,3
Retrognatism^a^	6	10,7	1,3
Low-set ears (Q17.4)	6	10,7	1,3
Exophtalmia (=Macrophtalmos) (Q11.3)	3	5,35	0,7
Microphtalmos (Q11.2)	1	1,8	0,2
Congenital glaucoma (Q15.0)	1	1,8	0,2
Frontal bossing^a^	1	1,8	0,2
congenital absence of auricle (Q16.0)	1	1,8	0,2
Canines^a^	1	1,8	0,2
Gum tooth^a^	1	1,8	0,2
Total	56	100.00	12,7
**Q20-Q28 Congenital malformations of the circulatory system**			
Congenital valve malformation^a^	10	26,31	2,2
Anasarca fetoplacental^a^	7	18,42	1,6
Cardiomegaly^a^	5	13,15	1,1
Cystic hygroma^a^	5	13,15	1,1
Single umbilical artery (Q27.0)	4	10,52	0,9
Hydrothorax^a^	2	5,3	0,45
Laevocardia (Q24.1)	1	2,63	0,2
Tetralogy of Fallot (Q21.3)	1	2,63	0,2
Congenital malformation of heart, unspecified (Q24.9)	1	2,63	0,2
Single umbilical artery (Q27.0)	1	2,63	0,2
2 arteries + 2 veins^a^	1	2,63	0,2
Total	38	100.00	8,6
**Q30-Q34 Congenital malformations of the respiratory system**			
Agenesis of nose cartilage (Q30.1)	2	40	0,45
Hypoplasia and dysplasia of lung (Q33.6)	2	40	0,45
Agenesis of lung (Q33.3)	1	20	0,2
Total	5	100.00	1,1
**Q35-Q37 Cleft lip and cleft palate**			
Cleft palate with cleft lip (Q37.-)	9	42,86	2
Cleft lip (Q36.-)	7	33,33	1,6
Cleft palate (Q35.-)	3	14,3	0,6
Alveolar cleft^a^	1	4,76	0,2
Velopalatine cleft ^a^	1	4,76	0,2
Total	21	100.00	4,8
**Q38-Q45 Other congenital malformations of the digestive system**			
Atresia of oesophagus NOS (Q39.0)	6	33,33	1,3
Hepatomegaly (Q44.7)	3	16,7	0,6
Imperforate anus (Q42.3)	2	11,11	0,45
Antal malformation (Q43.9)	2	11,11	0,45
Congenital malformation of mouth NOS (Q38.6)	1	5,55	0,2
High arched palate (Q38.5)	1	5,55	0,2
Macroglossia (Q38.2)	1	5,55	0,2
Glossoptosis ^a^	1	5,55	0,2
Congenital dilatation of the colon (Q43.9)	1	5,55	0,2
Total	18	100.00	4,1
**Q50-Q56 Congenital malformations of genital organs**			
Hypospadias (Q54.9)^a^	7	28	1,6
Micropenis (Q55.6)	7	28	1,6
Ambiguous genitalia (Q56.4)	6	24	1,3
Cryptorchism (Q53.9)	3	12	0,6
Invisible clitoris (Q52.6)	1	4	0,2
Congenital malformation of female genitalia (Q52.9)	1	4	0,2
Total	25	100.00	5,7
**Q60-Q64 Congenital malformations of the urinary system**			
Polycystic kidney (Q61.3)	2	20	0,45
Ureterohydronephrosis (Q62.-)	2	20	0,45
Epispadias (Q64.0)	2	20	0,45
Absence of bladder (Q64.5)	2	20	0,45
Congenital dilatation of ureter (Q62.2)	1	10	0,2
Congenital displaced kidney (Q63.2)	1	10	0,2
Total	10	100.00	2,27
**Q65-Q79 Congenital malformations and deformations of the musculoskeletal system**			
Clubfoot (Q66.8)	27	17,4	6
Omphalocele (Q79.2)	12	7,7	2,7
Accessory fingers (Q69.0)	11	7,1	2,5
Reduced limbs (Q73.8)	10	6,4	2,3
Gastroschisis (Q79.3)	8	5,1	1,8
Chondrodysplasia punctata (Q77.3)	6	3,9	1,3
Talipes equinovarus (Q66.0)	5	3,2	1,1
Macrocephaly (Q75.3)	5	3,2	1,1
Prune Belly syndrome (Q79.4)	5	3,2	1,1
Syndactyly (Q70.-)	4	2,6	0,9
Toe agenesis^a^	4	2,6	0,9
Feet valgus (Q66.6)	3	1,9	0,7
Clinodactyly^a^	3	1,9	0,7
Fingers agenesis^a^	3	1,9	0,7
Congenital anomaly of limb(s) (Q74.9)	3	1,9	0,7
Thanatophoric dysplasia^a^	3	1,9	0,7
Craniosynostosis (Q75.0)	3	1,9	0,7
Hypertelorism (Q75.2)	2	1,3	0,4
Hyperlaxity ligament^a^	2	1,3	0,4
Congenital absence of limbs (Q73.0)	2	1,3	0,4
Forearm agenesis^a^	2	1,3	0,4
Foot agenesis^a^	2	1,3	0,4
Limbs in hyperflexion ^a^	2	1,3	0,4
Limbs asymmetry^a^	2	1,3	0,4
Congenital dislocation of hip (Q65.2)	2	1,3	0,4
Scoliosis (Q67.5)	2	1,3	0,4
Caudal regression syndrome^a^	2	1,3	0,4
Diaphragmatic hernia (Q79.0)	2	1,3	0,4
Narrow thorax (Q67.8)	2	1,3	0,4
Amniotic bands^a^	1	0,64	0,2
Microdactyly^a^	1	0,64	0,2
Hand agenesis^a^	1	0,64	0,2
Phalanges agenesis^a^	1	0,64	0,2
Thumb hypoplasia^a^	1	0,64	0,2
Club hand^a^	1	0,64	0,2
Bone growth^a^	1	0,64	0,2
Spine agenesis^a^	1	0,64	0,2
Sarcum agenesis^a^	1	0,64	0,2
Parieto-occipital bone agenesis^a^	1	0,64	0,2
Congenital funnel chest (Q67.6)	1	0,64	0,2
Malformation of ribs (Q76.6)	1	0,64	0,2
Thin thorax (Q67.8)	1	0,64	0,2
Asymetric thorax (Q76.9)	1	0,64	0,2
Deformed thorax (Q76.9)	1	0,64	0,2
Expanded Thorax (Q67.8)	1	0,64	0,2
Total	155	100.00	35
**Q80-Q89 Other congenital malformations**			
Ichtyosis vulgaris (Q80.0)	3	16,7	0,7
Cervico-facial hemolymphangioma^a^	2	11,11	0,4
Congenital splenomegaly^a^	2	11,11	0,4
Alopecia (Q84.0)	1	5,55	0,2
Naevus (Q82.5)	1	5,55	0,2
Epidermal sinus^a^	1	5,55	0,2
Depigmentation^a^	1	5,55	0,2
Situs inversus (Q89.3)	1	5,55	0,2
Umbilical cord membrane detachment^a^	1	5,55	0,2
Pierre Robin syndrome (Q87.0)	1	5,55	0,2
Acardiac fetus^a^	1	5,55	0,2
Absence of gluteal fold^a^	1	5,55	0,2
Cyclopia (Q87.0)	1	5,55	0,2
Ombilical hernia^a^	1	5,55	0,2
Total	18	100.00	4
**Q90-Q99 Chromosomal abnormalities**			
Down syndrome (=Down syndrome) (Q90.-)	35	87,5	8
Trisomy 13 (Q91.7)	2	5	0,4
Trisomy 18 (Q91.3)	1	2,5	0,2
Trisomy 8 (Q92.1)	1	2,5	0,2
Senior Loken syndrome^a^	1	2,5	0,2
Total	40	100.00	9

*No* Number of cases, % percentage

^a^Congenital malformations not found in the ICD-10 classification

Malformation diagnosis was performed in more than two thirds of cases (71.4%) at birth during a systematic newborn infant’s clinical examination.

The antenatal diagnosis, which represents 28.6% of cases, was performed mainly in the second and third trimesters with a proportion of respectively 47% (33 cases) and 48.5% (34 cases). The ultrasound diagnosis during the first trimester only covered 5.7% (4 cases).

The factorial analysis has identified a seven-factor structure (associations of malformations) accounting for 21.28% of the overall variance (Table 3). The first three associations accounted for more than 50% of the total variance.

- The first factor is an association of congenital malformations corresponding to Trisomy 8, pelvic kidney, microphthalmia, sacral agenesis, epidermal sinus, agenesis of nose cartilage and clubfoot.
- The second factor is an association of congenital malformations corresponding to craniosynostosis, funnel chest, limb asymmetry, scoliosis, cleft palate and ureteral dilatation.
- The third factor is an association of congenital malformations corresponding to invisible bladder, absence of thalami, thin thorax, hyperflexion limbs, cardiomegaly, amniotic flanges and polycystic kidney.

**Table 3:** Analysis of congenital malformations profiles detected at the “Les Orangers” Maternity and Reproductive Health Hospital, Rabat, Morocco

Profile	Congenital malformations	% of variance	% cumulated
**1**	- Trisomy 8 - Ectopic kidney - Microphthalmia - Sarcum agenesis - Epidermal sinus - Agenesis of nose cartilage - Clubfoot	**4,62**	**4,62**
**2**	- Craniosynostosis - Funnel chest - Limbs asymmetry - Scoliosis - Cleft palate - Congenital dilatation of ureter	**3,36**	**7,98**
**3**	- Invisible bladder - Absence of thalami - Thin thorax - Hyperflexion of limbs - Cardiomegaly - Amniotic band - Polycystic kidney	**2,9**	**10,88**
**4**	- Thin thorax - Cranio-facial dysmorphia - Microdactyly - Short limbs	**2,8**	**13,68**
**5**	- Gingival hypertrophy - Macroglossia - Limbs malformation - Ichtyosis - Exophtalmia - Dandy Walker syndrome	**2,7**	**16,38**
**6**	- Acardiac fetus - Glossoptosis - Pierre Robin syndrome - Retrognatism	**2,5**	**18,88**
**7**	- Canines - Deformed thorax - Hypertelorism - Retrognatism - Low-set ears	**2,4**	**21,28**

% = percentage

## Discussion

In the present study, a prevalence of 5.58 per thousand births (0.56% or 55.8 per 10,000 births) was reported. This prevalence is consistent with that of other African countries.

Indeed, a retrospective analytical study carried out in the city of Lubumbashi, Congo between 2010 and 2011 across 11 maternity clinics showed a prevalence of 58.4 per 10,000 births (0.58%) [13]. A similar prevalence (0.57%) was also found in a multicenter prospective study conducted at Clinical Universities of Kinshasa [14].

In addition, a study conducted in Egypt between 1995 and 2009 showed a frequency of CMs of 2% [15].

In developed countries, where congenital anomalies are systematically reported in national registries, the prevalence of congenital malformations is 6 times higher than the one we have reported in our study. In France, for example, the estimated prevalence of congenital malformations at birth is 3–4% [16] while it stands at 3–5% in Canada and the United States [17, 18].

Thus, the prevalence reported in our study is probably below the real value due to possible under-reporting of malformations in addition to the fact that some late-onset malformations, posterior to neonatal period, were not accounted for. This observation is found in a majority of studies carried out in developing countries [19, 20].

The most frequently reported malformations in our series were musculoskeletal abnormalities, CMs of nervous system and CMs of eye, ear, face and neck. These 3 types of malformations represent 60% of reported cases.

Musculoskeletal abnormalities were the most frequently reported with a rate of 33% (equivalent to 35 per 10,000 total births), of which 17.4% of clubfoot, 7.7% of omphalocele, 7.1% of supernumerary fingers, followed by short limbs with a rate of 6.4%, and laparoschisis 5.1%.

The predominance of musculoskeletal abnormalities was also reported in other countries [21, 22].

A study by Sakar et al. in India reported a similar prevalence of musculoskeletal abnormalities at 33% followed by abnormalities of digestive and central nervous systems [23].

In Egypt, the rate of musculoskeletal abnormalities is 8.82% (1.8 / 1000) [15].

A study by Dolk et al. in Europe indicated that limb abnormalities were more frequent (38 per 10,000 births) than neurological one (23 per 10,000 births) [24].

In Canada, the prevalence of limb malformations was estimated at 3.5 per 10,000 total births in 2007, and the prevalence of laparoschisis was 4.4 per 10,000 total births in 2009 [17].

CM of the nervous system was the second most reported in our study with a percentage of 18% (19 per 10,000 births). Out of these reported cases, 31% were of hydrocephalus (5.9 per 10,000 total births), 26.2% of anencephaly (versus 5 per 10,000 total births), 20.2% spina bifida (3.8 per 10,000 total births).

However some studies, such as those in Irak, India, Turkey and Ethiopia [20, 25–27], reported that neurological malformations ranked first with higher prevalence.

Similarly, a study conducted in Egypt between 1995 and 2009 reported a higher prevalence of neurological malformations at a rate of 55/10,000 and a lower prevalence of musculoskeletal abnormalities at 18/10,000 [15].

These differences in reported prevalence rates between studies reflect significant differences between populations, the environment and health policies of each country. They also reflect difference in collection period, recruitment mode or definition of congenital malformation cases.

In our study, the prevalence of neural tube defects is reported at 10 per 10,000 total births. This prevalence varies between countries from a low 4.1 per 10,000 births in Canada [17] to a high 12.8 per 10,000 births in England and Wales [28] while, in the European Union, the current prevalence of neural tube defects (NTD) was estimated at 10.8 (9.80–10.36) per 10,000 births between 2011 and 2017 according to the latest results published by EUROCAT [29].

The prevalence remains high in developing countries while it has gone down significantly in countries which implemented NTD prevention policies including folic acid supplementation and prenatal NTD screening and pregnancy interruption for severe cases [17].

In Morocco, the ministry of health started a folic acid supplementation strategy in 2008 according to a protocol which specifies a supplementation of 400 μg of vitamin B9 to parturient women while for epileptic under-treatment women (sodium valproate, carbamazepine) and women with a history of neural tube closure abnormalities (an affected newborn or family cases) the protocol specifies a 5 mg/day dose 2 months prior to conceiving and during the first 3 months of pregnancy.

Genetic abnormalities represent 8.5% (9 per 10,000 total births), 87.5% of which are Down syndrome (8 per 10,000 total births). In Egypt, these genetic abnormalities represent 25% (5,1/1000 total births), 74.49% of which are Down syndrome [15].

The prevalence of Down syndrome in our study is lower than those reported by others. Indeed, A study conducted in the Rhône-Alpes region over the 1981–2009 period showed a prevalence of 28.7 per 10,000. In addition, a study covering the population of Paris region between 1981 and 2007 estimated the total prevalence of Down syndrome at 30.6 per 10,000 while the prevalence among live births was 8.9 per 10,000; a difference is mainly due to prenatal screening and medical termination of pregnancy (MTP).

The high prevalence of Down syndrome in developed country is attributable to the advanced maternal age at procreation and early screening policies [30].

The screening for Down syndrome is not generalized in Morocco which does not have a chromosomal screening policy in place. In addition, pregnancy interruption is not legally permitted.

The antenatal screening for congenital malformations is not systematic in developing countries, including Morocco, as it is not integrated in national health programs. This explains the low rate of antenatal diagnosis in our study which stands at 28.6% of cases, of which 5.7% (4 cases) were diagnosed in the 1st trimester, 47% (33 cases) in the 2nd trimester and 48.5% in the 3rd trimester.

The antenatal diagnoses performed during the 3rd trimester a related to pregnant women who were initially under the care of other health structures where no early screening was carried out, in addition to women who only sought medical care when they reached their 3rd trimester.

This delay in antenatal diagnosis of congenital malformations is also reported in other studies covering developing countries with, for example, a median gestational age of 31 weeks in Saudi Arabia and 32 weeks in Kenya [31, 32].

In more than two thirds of cases (71.4%), the diagnosis was made at birth during the systematic clinical examination of newborn infants.

In our study, 26.5% of births with malformation presented a polymalformative syndrome. Seven groups of malformation associations were identified through principal component analysis. These associations can explain 21% of the malformation variability in the studied population. The 3 main associations represent more than 50% of the total variance. These 3 associations include, among other malformations, the VACTERL/VATER combination, a group of congenital malformations including vertebral “V”, anorectal “A”, cardiovascular “C”, tracheo-esophageal “TE”, renal “R” and limbs “L”.

The diagnosis of VACTERL association can only be confirmed once at least 3 of the above-mentioned congenital malformations are identified in a patient [33, 34].

### Study limitations

This study allowed us to estimate the prevalence of congenital malformations in the case of Morocco. However, this result is probably understating the reality of the national epidemiological situation since the malformation census is not systematically performed throughout the country. In addition, health programs have not integrated routine prenatal screening for CM and do not consider this problem a priority at this time. Moreover, some late-onset malformations, posterior to neonatal period, will not be counted in the census.

Finally, the insufficient description of some congenital malformation cases lead to a general under-reporting of malformations.

## Conclusion

The present study evaluated the prevalence of CMs at the “Les Orangers” Maternal and Reproductive Health Hospital in Rabat, and determined the different types and existing associations of these malformations between January 1st 2011 and June 31st 2016.

It represents an epidemiological database that should be complemented by other multicenter studies to determine the prevalence of congenital malformations at a national level.

Indeed, it is a imperative to ensure the monitoring of CMs and the accurate evaluation of their general prevalence and distribution in order to assess the burden on the population as well as the health system and select the appropriate preventive measures.

Furthermore, the registration and monitoring of CMs in our country should be established as a whole program including a congenital anomalies registry as part of the national health information system and a teratovigilance network. This will allow to consider a national and/or regional strategy for antenatal diagnosis, genetic counseling and prevention of CMs. Additionally, it will enable a better management of newborns with congenital anomalies from birth.

## Data Availability

Data is available upon reasonable request.

## Abbreviations

CM: Congenital malformations
WHO: World health organization
ENPSF: National Survey of Population and Family Health
EUROCAT: European surveillance of congenital anomalies
ICBDSR: International clearinghouse for birth defects surveillance and research
VACTER/VATER: Acronym for vertebral, anal, cardiac, tracheal, esophageal, radial, and/or renal malformations
PCA: Principal component analysis
FDIU: Fetal death in-utero
CDC: Centers for Disease Control and Prevention
NTDs: Neural tube defects
MTP: Medical termination of pregnancy
CI: Confidence interval
ICD: International classification of diseases
